# Fabrication of Polymer Microstructures of Various Angles via Synchrotron X-Ray Lithography Using Simple Dimensional Transformation

**DOI:** 10.3390/ma11081460

**Published:** 2018-08-17

**Authors:** Kyungjin Park, Kanghyun Kim, Seung Chul Lee, Geunbae Lim, Jong Hyun Kim

**Affiliations:** 1 School of Interdisciplinary Bioscience and Bioengineering, Pohang University of Science and Technology (POSTECH), 77 Cheongam-ro, Nam-gu, 37673 Pohang, Korea; kjpark@postech.ac.kr; 2 Department of Mechanical Engineering, Pohang University of Science and Technology (POSTECH), 77 Cheongam-ro, Nam-gu, 37673 Pohang, Korea; qwnerkang@postech.ac.kr; 3 Pohang Accelerator Laboratory (PAL), Pohang University of Science and Technology (POSTECH), 77 Cheongam-ro, Nam-gu, 37673 Pohang, Korea; leesch00@postech.ac.kr

**Keywords:** Synchrotron X-rays, lithography, curved substrate, microstructures

## Abstract

In this paper, we developed a method of fabricating polymer microstructures at various angles on a single substrate via synchrotron X-ray lithography coupled with simple dimensional transformations. Earlier efforts to create various three-dimensional (3D) features on flat substrates focused on the exposure technology, material properties, and light sources. A few research groups have sought to create microstructures on curved substrates. We created tilted microstructures of various angles by simply deforming the substrate from 3D to two-dimensional (2D). The microstructural inclination angles changed depending on the angles of the support at particular positions. We used convex, concave, and S-shaped supports to fabricate microstructures with high aspect ratios (1:11) and high inclination angles (to 79°). The method is simple and can be extended to various 3D microstructural applications; for example, the fabrication of microarrays for optical components, and tilted micro/nanochannels for biological applications.

## 1. Introduction

Synchrotron X-ray lithography (XRL) uses a hard X-ray source to fabricate nano/microstructures of various shapes [1,2]. Synchrotron XRL has been used to create high aspect ratio structures with good sidewall roughness; synchrotron X-rays are highly collimated, pass through thick polymers and other plastic materials, and exhibit high flux and intensity, the pattern accuracy of XRL is very high. Synchrotron XRL was first developed to fabricate uranium-separation nozzles, and several other applications have been found since. For example, synchrotron XRL has been used to fabricate high-density magnetic dot arrays [3], a vertical stepper [4], and micromechanical components [5].

Ultraviolet (UV) light is employed in conventional photolithography. The photomask pattern is copied to photoresist (PR) with various ratios, and a two-dimensional (2D) microstructure is formed on a flat substrate. Various techniques are used to create three-dimensional (3D) features on 2D substrates. Micro stereo lithography [6], 3D printing [7], oblique lithography [8], PR reflow [9], backside exposure lithography [10], grayscale lithography [11], nanoimprint lithography [12,13], nanosphere lithography [14], colloidal lithography [15], block copolymer lithography [16], and e-beam lithography [17] all allow extensive shaping. However, it is complicated to fabricate the desired structures using these techniques.

In 2011, Jongho et al. prepared copper microstructures on curved substrates via optical soft lithography and metal electroplating [18]. The microstructures had high aspect ratios, throughput was good, cost was low, and reproducibility was high.

Microcontact printing using cylindrical polydimethylsiloxane (PDMS) stamps has since been reported [19]. However, it was not possible to create various angles on a single substrate by flattening the curved substrates bearing the microstructures.

Here, we develop a method by which to fabricate tilted microstructures of various angles and shapes on a single substrate using synchrotron XRL. Synchrotron X-rays are highly collimated; they pass through polymers (because of their short wavelength). Such a unique feature of hard X-rays allows the fabrication of microstructures on curved substrates. The fabrication technique on curved substrates could be applied to the fabrication of precision optical elements [20] and the micromachining of ultraprecise molds on curved or cylindrical structures.

## 2. Materials and Methods

### 2.1. Experimental Setup

**Figure 1:**
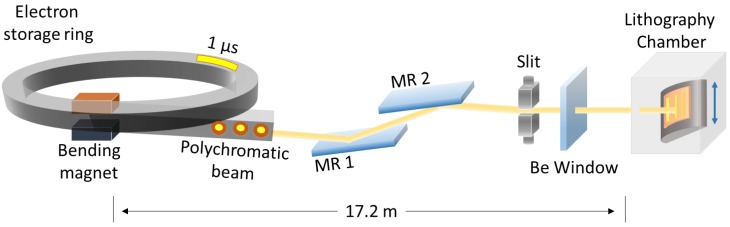
Schematic of synchrotron X-ray lithography. MR, mirror; Be, beryllium.

The experiment was conducted at the 9D (X-ray nano/micromachining [XNMM]) beamline [21] of the Pohang Light Source (PLS) using a polychromatic X-ray beam (Figure 1) emitted from an electron storage ring. Unlike conventional UV lithography, it is possible to impart dimensional changes to the X-ray mask to create gaps between the mask and the sample, thus improving technical performance.

The energy of the linearly accelerated electrons is 3 GeV, and the current moving around the storage ring is 400 mA (Table 1). The 9D beamline uses a bending magnet to direct the X-rays into the lithography chamber. The horizontal divergence of the beam source from the bending magnet is about 8 mrad (Table 1). As the distance from the magnet to the sample is about 20 m, the beam width is about 16 cm, which is sufficient for a four-inch wafer process. X-rays emitted from the magnet pass through two mirrors and the Be windows before finally reaching the lithography chamber. The mirrors are inserted in the beam path to remove high-energy emissions [22]. The Be windows isolate the vacuum and experimental zones, and are surrounded by He (an inert, non-toxic noble gas). The He gas prevents Be window oxidation. The experimental chamber is also filled with He because the gas exhibits good X-ray transmittance, and flux degradation is minimal. Another effect of He atmosphere is that He atmosphere promotes heat dissipation by convection in the chamber, since He has high heat capacity. The sample stage of the experimental chamber moves vertically to provide homogeneous X-ray exposure distribution. The beam width covers the entire sample in the horizontal direction, but the vertical beam dimension is only about 1 cm; thus, the sample stage must move vertically to cover the entire sample (Figure S1).

### 2.2. Fabrication of a Flexible X-Ray Mask

Synchrotron XRL was used to create microstructures of various angles via simple dimensional transformations. Especially when using a curved substrate, the mask must be sufficiently flexible to adhere to the PR.

**Figure 2:**
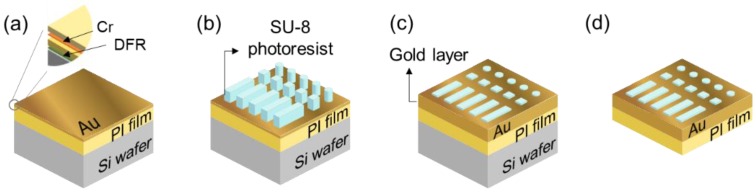
Fabrication of a flexible X-ray mask. (**a**) A gold layer on the polyimide (PI) film and silicon (Si) wafer; (**b**) Mask patterns on the gold layer after photolithography; (**c**) An electroplated gold (Au) layer between the mask patterns; (**d**) The completed flexible X-ray mask after separation from the Si wafer substrate. DFR, dry film resist.

Figure 2 shows the fabrication of the flexible X-ray mask. The first step was the deposition of a gold layer on the polyimide (PI) film/silicon (Si) wafer (Figure 2a). The PI film was laminated onto the Si wafer using dry film resist (DFR) as adhesive. The PI film/Si wafer was chemically prepared and oxygen plasma-cleaned; the film was chemically resistant and thermostable. After cleaning and surface treatment, a 20-nm chrome layer and an 100-nm gold layer were then deposited. The chrome layer served to adhere the PI and the gold. A 100-nm gold seed layer for electroplating is then deposited. The second step in mask formation is shown in Figure 2b. The PR on the PI film is formed via UV photolithography. To create the PR, the PR (SU-8 3010; MicroChem, Westborough, MA, USA) was spin-coated onto the gold layer at 1,000 rpm for 30 s, and the sample was heated for 8 min at 95 °C (the soft bake). When all of the wrinkles had been removed, the PR was covered by the chrome mask and exposed to 15 mW of UV for 12 s (total energy: 175 mJ/cm^2^), followed by post-exposure baking (PEB) at 65 °C for 1 min and 95 °C for 23 min, rendering the latent PR pattern visible.

The next step was the developmental process, after which only the pattern remains; other areas were chemically removed to expose the bottom gold surface, which acts as a seed layer for gold deposition during electroplating. Plasma ashing was used to remove residual PR. Electroplating (to form the masking pattern) was then performed (Figure 2c) using a customized electrochemical cell. The electrolyte solution was a gold preparation (SP Gold 2000; CNC Tech., Seoul, Korea). The gold substrate (the cathode) was covered with electroplating tape except in the patterned SU-8 area. DC current density of 1 mA/cm^2^ was delivered to an area of 2600 mm^2^ at 50 °C with mild agitation. The electroplating time was calculated using the experimental data, and was about 3 h at an electroplating rate of 4 μm/h, affording a final gold thickness of 12 μm. Then, the substrate was thoroughly rinsed with distilled water. Finally, the electroplated gold structures were detached (Figure 2d).

**Figure 3:**
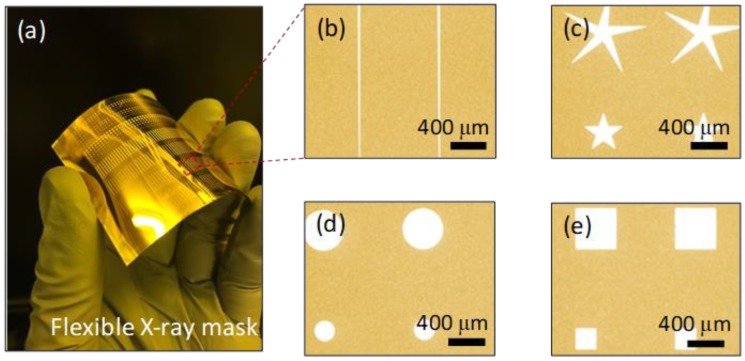
Flexible X-ray masks. (**a**) Picture of fabricated X-ray mask with various patterns including. (**b**) lines, (**c**) stars, (**d**) circles, (**e**) squares.

Optical microscopy confirmed that the SU-8 PR remained adherent to the substrate during mask fabrication. We created seven different patterns: four rows of lines with 20-μm, 50-μm, 100-μm, 200-μm, and 800-μm gaps between them; and rectangular, star, and circle patterns, as shown in Figure 3. Optical microscopy showed that the pattern edges were neatly formed, not only for relatively simple circles but also for (irregular) stars. We encountered no stress-induced gold peeling during electroplating.

### 2.3. Fabrication of Microstructures of Various Angles

In this research, PR SU-8, the material used for the microstructures, was micropatterned onto a flexible substrate, forming structures >100 μm in height. In fact, the PR exhibited excellent substrate adhesion, even when forming structures of height >3 mm via dry-chip casting [23]. Therefore, SU-8 is optimal for producing tall structures with various angles and shapes.

#### 2.3.1. Spin-Coating

**Figure 4:**
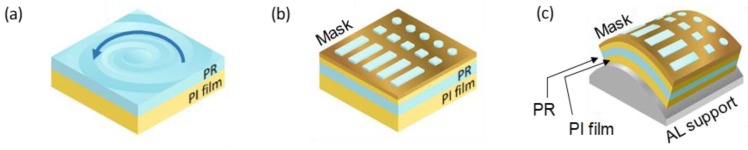

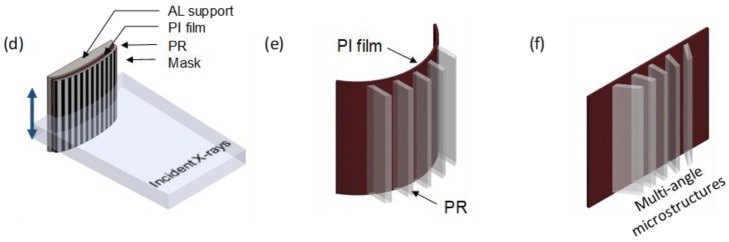
Fabrication of microstructures of various angles. (**a**) Spin-coating of photoresist (PR) onto a PI film. (**b**) The flexible PR X-ray mask. (**c**) The flexible structure wrapped on a curved substrate. (**d**) X-ray exposure of the curved structure. (**e**) Microstructures on a curved substrate. (**f**) Microstructures tilted at various angles.

After fabricating the mask, the SU-8 was first spin-coated onto a flat structure. Although spraying and roller-coating, dip-coating, and extrusion-coating methods are available, spin-coating is most commonly used to form flat PR layers. The height of the final structure was about 75% of the thickness of the PR. The micropatterned structure was supported by a flexible PI film. The PI film (5 cm × 4 cm) was prepared via chemical and plasma cleaning, followed by surface treatment, as described for mask fabrication. PR (SU-8 2075; MicroChem) was spin-coated at 1000 rpm to a thickness of about 120 μm (Figure 4a), followed by heating (soft bake) to remove the solvent and cure the PR. The soft bake was performed at 65 °C for 8 min and 95 °C for 45 min. Wrinkles could have formed if the heating had been inadequate, resulting in surface distortion on contact with the mask, and thus the creation of dents on removal of the mask. In addition, the development time would have been extended. Then, the flexible X-ray mask was attached on top of the PR (Figure 4b) using an intermediate 30-μm PI film to prevent sticking.

#### 2.3.2. X-Ray Exposure

The PR with the flexible X-ray mask was attached to a semicircular column by a lead sheet (Figure 4c), and then attached to a sample stage in the experimental chamber. Cooling lines were run under the sample stage. Heat control is important; heat generated by X-rays may deform the PR layer or create bubbles. After placing the sample in the stage, the chamber was evacuated and helium gas was injected. The X-ray beam dimensions were about 10 cm horizontally and 1 cm vertically. The stage had a reciprocating vertical motion to cover the entire field of view (FoV) of the sample (Figure 4d). The vertical scan speed was 2 cm/s and the total scan range was 7 cm. The dose delivered to the PR was about 90 J/cm^3^. After X-ray exposure, the PI/ PR construct was removed from the curved support, and PEB was performed at 65 °C for 5 min and 95 °C for 10 min. Irradiated, negative X-ray PRs form strong acids, and the affected areas become cross-linked during PEB. The brittleness and ductility of the photopolymer during exposure and PEB are influenced by the sensitivity of the PR to X-rays, X-ray intensity, exposure time, temperature, and PEB time; all must be controlled to ensure appropriate microstructural stiffness and adhesion to the flexible substrate.

#### 2.3.3. Development

After PEB, the PR/PI film was immersed in SU-8 developer for about 15 min. During development, the area exposed to the X-ray remains, and the area that is not exposed is removed (Figure 4e). As the substrate is flexible, it can be flattened, and the structures then exhibit various tilt angles depending on their distances from the center (Figure 4f).

## 3. Results

### 3.1. Fabricated Polymer Microstructures of Various Shapes on a Flexible Substrate.

**Figure 5:**
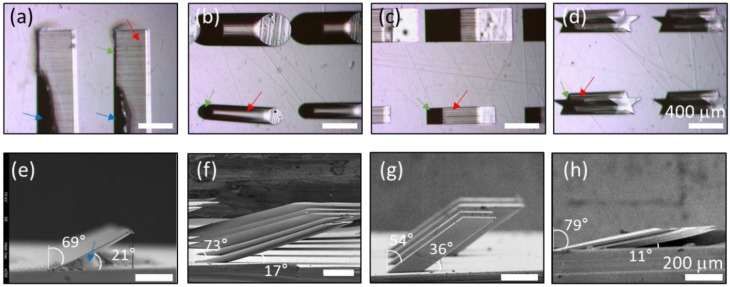
Fabricated polymer microstructures of various shapes on a flexible substrate. Optical microscopic images of various patterns (from the top) including (**a**) lines, (**b**) circles, (**c**) stars, (**d**) squares. Scanning electron microscopy (SEM) images of various patterns (side views): (**e**) lines, (**f**) circles, (**g**) stars, (**h**) squares. Scale bars, (**a**–**d**) 400 μm, (**e**–**h**) 200 μm.

Representative examples of the tilted microstructures are shown in Figure 5. Figure 5e–h are optical images acquired in top–down view; the shapes include lines, circles, stars, and squares. As the microstructural material is a polymer, the microstructures are transparent to visible light, allowing the morphology of the bottom substrate to be observed (red arrows in Figure 5a–d). The bottoms of the microstructures are dark (green arrows in Figure 5a–d). The four microstructures are shown from the side via scanning electron microscopy (SEM) in Figure 5e–h. The width of the line pattern microstructure is 50 μm; the length is 5 mm, the height is about 200 μm, the hypotenuse is about 560 μm, and the tilt angle is about 69°. The aspect ratio of the tilted line structure is about 1:11. As the pattern is narrow, long, and highly tilted, the structure is vulnerable to external forces. Especially, during development, the developer solution must be circulated to remove non-crosslinked SU-8 PR. If stirring is too strong, the structure will collapse. If stirring is too weak, some polymer will remain near the bottom, i.e., under the tilted structure (blue arrow in Figure 5e). In Figure 5a, residual polymer is present in the dark area (blue arrow). The radius of the smaller circular microstructure is 100 μm; the height is about 240 μm, the hypotenuse is 840 μm, and the tilt angle is about 73°. We also fabricated square-patterned microstructures with a tilting angle of 54°. The height of the structure was about 300 μm, the length of a side was about 200 μm, and the hypotenuse was 510 μm. Interestingly, we observed that star-patterned microstructures had the highest tilting angles, as shown in Figure 5h. The area of the star pattern was about 45 μm^2^, the height of the microstructure was about 100 μm, the hypotenuse was 510 μm, and the tilting angle was about 79°.

### 3.2. Fabricated Polymer Microstructures of Various Angles on a Flexible Substrate

**Figure 6:**
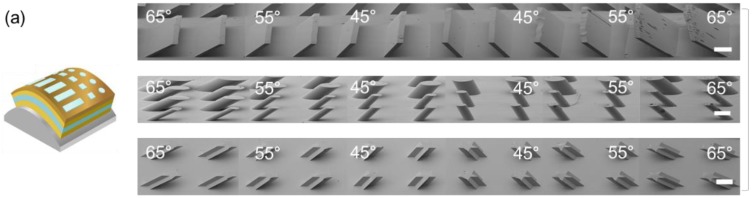

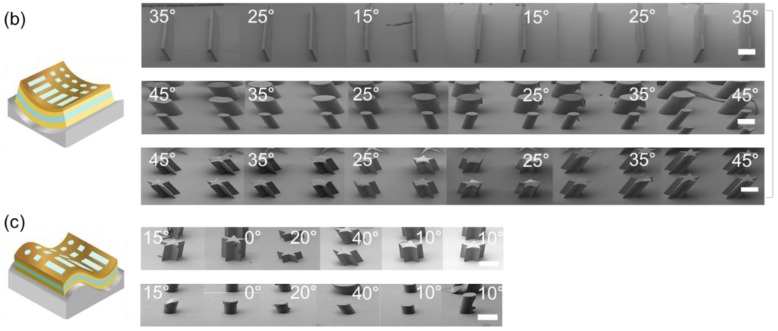
SEM images of microstructures fabricated on the (**a**) convex support, (**b**) concave support, (**c**) S-shaped support.

As shown in Figure 6, the polymer microstructure was fabricated on a single substrate with a single synchrotron X-ray exposure. The key to this development was that the tilt angle of the microstructures changed with the support structure. While the sample was irradiated with X-rays, the angle between the X-rays and the substrate of the microstructure determined the tilt angle of the microstructures. As shown in Figure 6a, when the exposure was performed with the PR attached to a convex support, the microstructures formed perpendicularly to the horizontal plane. When the substrate was flattened after separating the bent substrate from the convex support, the inclination angles of the microstructures were determined during exposure. For convex supports, the microstructures were inclined inwards at a greater angle as the position moved away from the center. Figure 6a shows a panoramic view of the tilted microstructures on a single substrate with tilt angles of 65°, 55°, and 45° as a representative data set. As shown in Figure 6a, the line, circle, and star microstructures had constant slopes at specific positions. For concave supports, the microstructures were inclined outwards at a greater angle as the position moved away from the center, as shown in Figure 6b. S-shaped supports were also used to fabricate microstructures on flexible substrates, as shown in Figure 6c. The S-shaped supports had two properties. The microstructures sloped inwards and outwards on the convex and concave supports, respectively.

One problem with the supports was that when a flexible X-ray mask was attached to the PR for exposure, some areas of the PR were pushed down when forces were applied to the flexible X-ray mask. In particular, it was difficult to attach the PR with a flexible X-ray mask on an S-shaped support because of its complicated structure. During the attachment, some deformation and damage occurred, and the height of the tilted microstructures varied at different positions. If slight deformation and damage occurred during attachment, the height of the tilted microstructures varied at different locations, as shown in Figure 6c.

**Figure 7:**
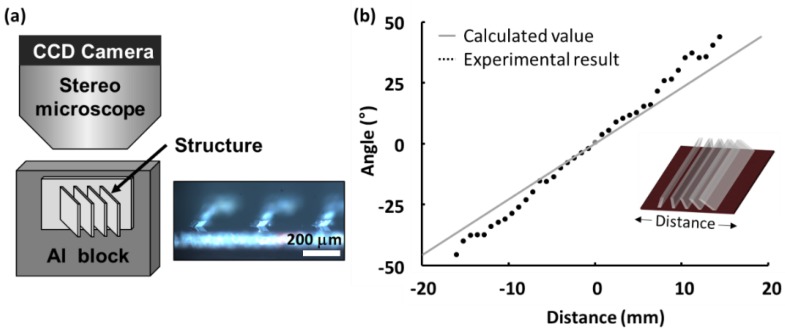
Angle measurement of the tiled microstructures. The structures were observed by a stereo microscope with a charge-coupled device (CCD) camera. (**a**) Experimental setup, (**b**) Calculated angle vs. measured angle.

We selected the line structure on the convex support to compare between the structures on the mask and the actual structures after the last processing step. The substrate of the tilted microstructures was attached to an aluminum block (Figure 7a). The block was placed on the stage of the stereo microscope (Stemi 2000-C, Carl Zeiss, Jena, Germany) with the lateral side up. The side images of the structures were recorded using a charge-coupled device (CCD) camera (Retiga 4000R; QImaging, Surrey, BC, Canada). A total of 39 angles were observed along a single row. The slanted angles were measured using the angle tool in the ImageJ software package (NIH, Bethesda, MD, USA). The measured angle was proportional to the position of the structure (Figure 7b). However, the distance from the center and the angle of the micropattern did not coincide exactly. The slopes from the experimental results were slightly higher than the calculated values due to detachment between the aluminum (AL) support and PI substrate. According to the angle measurement (Table S1), there was a gap in the center part, between the PI substrate and the support (Figure S3). When we placed the RP with the PI and mask on the Al support by hand, there was a slight air gap between the PI film and the Al support at the center.

## 4. Discussion

### 4.1. Interactions between the Beam Source and Materials

The mask on top of the PR layer blocked the X-rays selectively via gold patterned on a PI substrate; the PI film acted as a structural support that was transparent to X-rays.

**Figure 8:**
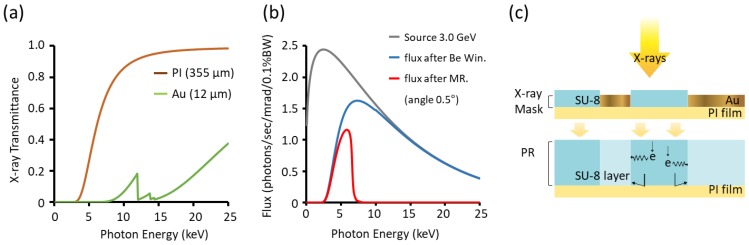
Experimental conditions for synchrotron X-ray lithography (XRL). (**a**) X-ray transmittance of the polyimide (PI) film and Au. (**b**) X-ray flux calculations before the beam entered the lithography chamber. (**c**) Schematic diagram of the exposure in the lithography chamber. PR, photoresist; MR, mirror; Be Win, beryllium window.

The micropatterns on the mask were made of SU-8 PR, which is transparent to X-rays. The background was a thick gold layer that absorbs X-rays; the SU-8 micropatterned area was selectively excluded from electroplating. The ratios of X-rays passing through gold and PI are shown in Figure 8a.

The flux of the beam on the mask was calculated by reference to the characteristics of the beam source, as shown in Figure 8b. Such analysis reveals not only the interactions between the electrons and the surrounding materials, but also the required gold thickness. We obtained a wide range of X-ray energies from the bending magnet, i.e., from a few volts to tens of kilo-electron volts (thus of both high and low energy). However, high-energy photons are detrimental, penetrating to the bottom of the PR and then scattering inappropriately into adjacent areas during X-ray exposure [22]. The schematic of Figure 8c shows a cross-section of the X-ray exposure in the lithography chamber. Such backscattered photons compromise structural accuracy and pattern/substrate adhesion. Also, backscattered photons that strike the edges of the structure damage those edges. Another problem associated with high-energy photons is secondary radiation. As photons pass through the PR, electrons emitted after photon absorption hit the walls under the gold mask (Figure 8c), thus degrading the wall. Therefore, mirrors were used to eliminate the high-energy photons with energies over 7 keV.

We calculated the thickness of the gold layer by calculating the X-ray flux and then estimating the dose incident on the PR numerically. The thickness of the gold was selected to minimize the transmission energy and was proportional to the product of the transmittance and the flux. The flux ejected from the linear accelerator fell by 66% after passing through the two Be windows (Figure 8b). The following equation [24] was used to calculate the number of transmitted X-rays (*S_TP_*):

(1) $$\;S_{TP}=\;S_{M}\;\times \exp\left(-\mu_{P}x_{P}\right)\;$$

where $x_{P}$ is the depth, $S_{M}$ is the X-ray energy spectrum of the PR, and $\mu_{P}$ is the absorption coefficient. The bottom doses should be about 90 J to promote cross-linking conditions. The thickness of the gold layer was calculated to be 10 μm. Thus, the SU-8 thickness must be >10 μm. The thickness was thus set to 12 μm, which was about 120% of the target thickness, with some margin.

### 4.2. The Utility of the Fabricated Structures in Real Applications

Again, we electroplated the microstructures with gold to make a mold for replicating the structure (Figure S4). We conducted a simple feasibility test to determine whether this mold can be used to fabricate microarrays for optical components, and tilted microchannels for many industrial applications. Further, the proposed development could also be applied to the manufacture of flexible electronics.

### 4.3. Limitations of the Fabrication Process

In the process described in Figure 4, both the substrate and the mask were bent. Due to the synchrotron beam being parallel across the entire FoV, there were deviations in the geometry over the whole FoV. There was a masking effect that resulted in a decreased structural width and increased structural height at the edge of the FoV. For 45° inclined structures, structures with a width of less than 12 µm were no longer illuminated by X-rays that did not partly pass through the gold absorber, thus reducing the dose absorbed by the resist. Theoretically, it is not possible to fabricate microstructures with critical dimensions less than the height of the mask pattern. The structures in this research were from tens of microns to a few hundred microns in height, so this effect was not obvious. This was not visible in the optical micrograph pictures of structures with a width of several 100 µm (Figure S2). However, it was of major relevance for the fabrication of structures smaller than the height of the mask pattern (12 μm).

The process can be modified to overcome this hindrance. For instance, the mask can be flat, and there can be a gap between the mask and the PR. In this case, the distance between the structures needs to be calculated exactly to achieve the desired pitch on the bending structure. In this research, we focused on providing a proof of concept and demonstrating the feasibility of using a simple dimensional transformation to produce polymer microstructures of various angles and shapes on a single substrate.

## 5. Conclusion

In this paper, we proposed a method of fabricating polymer microstructures with various angles on a single substrate by a simple dimensional transformation using synchrotron X-ray lithography. In the past, various techniques have been attempted to obtain various features on a 2D substrate, focusing on the exposure technology, material properties, and light source. Few research groups have attempted to create microstructures on 3D substrates. In this research, by the simple dimensional transformation of the substrate, we created tilted microstructures at various angles. Due to the high collimation of the synchrotron X-rays, microstructures with high aspect ratios could be realized simultaneously. The inclination angle of the microstructure changed depending on the support structure, which was at a constant inclination at each specific position. Three different-shaped supports: convex, concave, and S-shaped, were used to fabricate the microstructures. The microstructures had high aspect ratios of 1 to 11 for the line-shaped structures, and high inclination angles of 80° for the star-shaped structures. Further, the inclination angle of the microstructure can also be controlled by adjusting the degree of bending of the flexible substrate.

The developed method is simple, but can be extended to various microstructure applications. As a typical application, this technology can be applied to microarrays for optical components, and tilted micro/nanochannels for biological applications.

## Figures and Tables

**Table 1:** Specifications of the Pohang Light Source 9D nano/micromachining beamline.

Specification	9D Beamline
Electron energy	3 GeV ^1^
Beam current	400 mA
Horizontal beam span	8 mrad
Vertical beam span	0.34 mrad
Beam size at sample	100 mm (H) × 10 mm (V)

^1^http://pal.postech.ac.kr.

## Supplementary Materials

The following are available online at http://www.mdpi.com/1996-1944/11/8/1460/s1, Figure S1: Experimental chamber, Figure S2: Photograph of the microstructure, Figure S3: Schematics of attachment of PI film on the Al support, Figure S4: Fabrication process of the electroplated mold. Table S1. Sampled measured angle, measured position of the microstructures, and the calculated position.
