# Supplementary material for: Fabrication of Polymer Microstructures of Various Angles via Synchrotron X-Ray Lithography Using Simple Dimensional Transformation
Source: Materials (Basel). 2018 Aug 17;11(8):1460. doi: 10.3390/ma11081460 (PMC6119901; doi:10.3390/ma11081460)
Supplement: Supplementary file 1 [file materials-11-01460-s001.pdf]

materials

Supplementary Materials

Fabrication of Polymer Microstructures of Various Angles via Synchrotron X-Ray Lithography Using Simple Dimensional Transformation

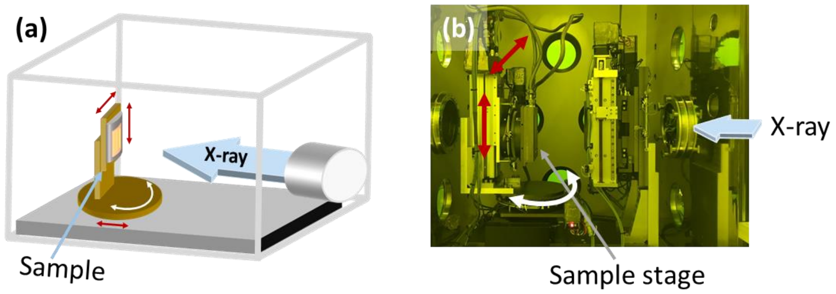

Figure S1. Experimental chamber. (**a**) Schematics of the experimental chamber, (**b**) Picture of the experimental chamber.

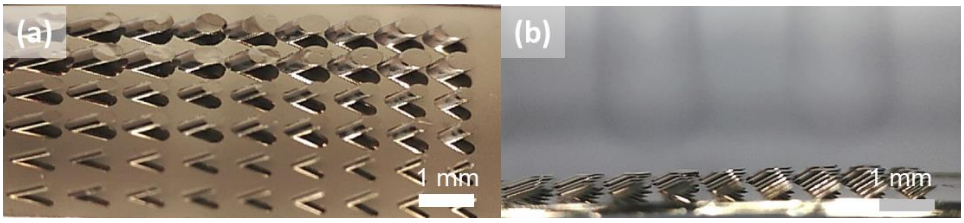

Figure S2. Photograph of the microstructure. (**a**) View from 45°, (**b**) View from almost 90°.

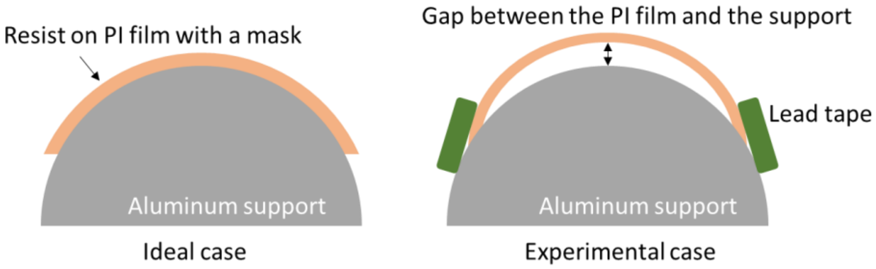

Figure S3. Schematics of attachment of PI film on the Al support.

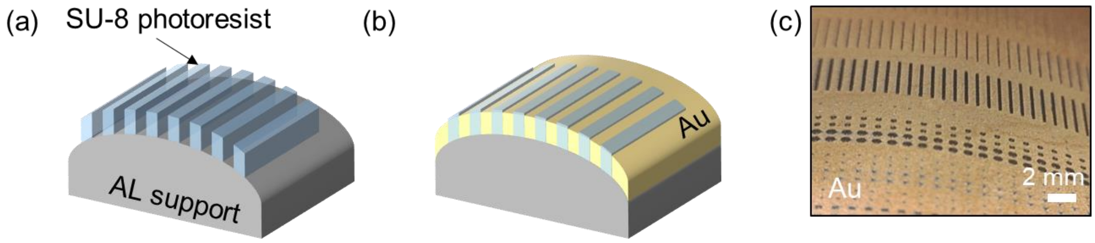

Figure S4. Fabrication process of the electroplated mold. (**a**) Schematic of the tilted microstructure on the PI film, (**b**) Schematic of the Au electroplating on the PI film, (**c**) Picture of the electroplated mold with tilted microstructures.

Table S1. Sampled measured angle, measured position of the microstructures, and the calculated position.

**Measured Angle (°)**	**43.9**	**26.0**	**5.6**	**−13.6**	**−32.1**	**−45.6**
Measured position (mm)	14.4	8.0	1.6	−4.8	−11.2	−16.0
Calculated position (mm)	19.2	11.3	2.5	−5.9	−14.0	−19.9

© 2018 by the authors. Submitted for possible open access publication under the terms and conditions of the Creative Commons Attribution (CC BY) license (http://creativecommons.org/licenses/by/4.0/).
